# Exploring workplace-based learning in distributed healthcare settings: a qualitative study

**DOI:** 10.1186/s12909-024-05053-6

**Published:** 2024-01-22

**Authors:** Myrthe J. M. Verhees, Anneke M. Landstra, Rik Engbers, Jur J. Koksma, Roland F. J. M. Laan

**Affiliations:** 1 Radboudumc Health Academy, Radboudumc, Gerard van Swietenlaan 2, Nijmegen, 6525 GB the Netherlands; 2 Rijnstate, Arnhem, the Netherlands

**Keywords:** Broker role, Clinical education, Curricular reform, Distributed medical education, Qualitative research methods, Workplace-based learning, Workplace learning

## Abstract

**Background:**

Distributed healthcare settings such as district hospitals, primary care, and public health facilities are becoming the real-life settings for workplace-based learning required to educate the future healthcare workforce. Therefore, a major focus should be on designing and developing workplace-based learning in these learning environments. Healthcare professionals and educational policymakers play a significant role in these settings as role models in workplace-based learning, and as leaders in integrating learning into their work environments. It is relevant to explore their beliefs, attitudes, and behaviors towards workplace-based learning in their own settings, in order to provide context-relevant recommendations that can assist in shaping workplace-based learning environments.

**Methods:**

We used individual interviews to understand professionals’ experiences with workplace-based learning in distributed healthcare settings. We - three clinicians, an educationalist, and a philosopher - thematically analyzed transcripts of 13 interviews with healthcare professionals and educational policymakers from different healthcare settings who were involved in the clinical phase of undergraduate medical education.

**Results:**

Clustering and categorizing of the data led to the construction of five overarching themes: Identification with and attitude towards medical education, Sense of ownership, Perceived time and space, Mutual preconceptions and relations, and Curriculum for a changing profession.

**Conclusions:**

These themes accentuate aspects relevant to the development of workplace-based learning in distributed healthcare settings on the individual, team, or organizational level. We highlight the significance of individual professionals in the development of workplace-based learning and emphasize the need for recognition and support for those occupying the ‘broker’ role at the intersection of education and practice. For future research and educational practice, we recommend prioritizing initiatives that build on good-practices in workplace-based learning and involve dedicated individuals in distributed healthcare settings.

**Supplementary Information:**

The online version contains supplementary material available at 10.1186/s12909-024-05053-6.

## Background

For over a decade now, ever since the plea made by Frenk et al. in 2010, medical schools are trying to reform their curricula to prepare future physicians for a constantly changing profession [1]. Constant change requires professionals to be adaptive and flexible life-long learners [2]. They should be competent to work collaboratively in patient-centered health systems [1]. At the curricular level, a transition from classroom-based to workplace-based learning seems inevitable for meeting these demands. After all, workplace-based learning provides fundamental learning opportunities for (future) healthcare professionals: learning in real life practices stimulates professional identity formation and a reflective attitude [3, 4].

As healthcare shifts towards a more patient-centered approach, care is increasingly being delivered in healthcare settings such as district hospitals, primary care clinics, and public health facilities [5, 6]. This development is also reflected in medical education, where these healthcare settings are increasingly recognized as valuable training sites for medical students (referred to as distributed medical education, DME) [7–10]. These workplace-based learning environments expose students to diverse patient populations and types of healthcare settings, and provide unique learning opportunities for learning about social determinants of health and integrated care [10–13]. In this paper, we use the term distributed healthcare settings to refer to healthcare settings other than the central academic hospital, such as district (teaching) hospitals, ambulatory care settings, and long-term care facilities.

Though studies report on the implementation and spread of DME on an overarching level, we have as yet limited insight in workplace-based learning in distributed settings [9, 10]. Workplace-based learning in distributed healthcare settings does not automatically fulfill its full potential with placing students in such settings: it is a complex process that we know is influenced by the working environment, student characteristics, supervisor characteristics, and educational characteristics in the workplace [10, 14, 15]. We know from literature that the workplace is often primarily focused on working rather than on learning [16, 17]. Also, when focused on learning, professionals tend to associate their own learning with formal continuous professional development systems, and not so much with their everyday work [16, 18].

It is precisely learning from this everyday work through role modeling that defines workplace-based learning, and that offers new and unique opportunities for students in DME settings. From literature, we know that educators’ leadership and motivation in teaching and learning influences workplace-based learning quality [19, 20]. However, in a questionnaire study, we recently found that healthcare professionals in different distributed healthcare settings indicated that, even though they expected their own institution to become more involved in medical education as a consequence of developments in the healthcare landscape, they did not foresee themselves getting more involved in it [21]. The challenges described in literature in designing workplace-based learning through the involvement of educators in University Medical Centers (UMCs) may even apply more to DME settings, with an even more pressing focus on production. Additionally, professional role models in DME settings are themselves likely to have been trained in traditional settings that differ from their current work environments. Therefore, they may have little experience with the dynamics of workplace-based learning in settings similar to their own, and their role in workplace-based learning might have had little explicit attention.

In this research, we interview professionals in distributed healthcare settings about workplace-based learning in their own work contexts. We aimed to answer the following research question: *What are the beliefs, attitudes, and behaviors of professionals concerning workplace-based learning in distributed healthcare settings?* We hope to provide context-relevant and concrete insights, which can help develop workplace-based learning in distributed healthcare settings. At the same time, we hope to inspire our participants to further shape and deepen their important role in transforming their workplace for the benefit of workplace-based learning.

## Methods

### Context and design of the study

Our research context was the clinical phase of undergraduate medical education in the Netherlands. Undergraduate medical students in the Netherlands follow a 6-year program, divided into a 3-year pre-clinical phase and a 3-year clinical phase. During the clinical phase, students rotate through various clerkships, which are workplace-based learning experiences that take place in different healthcare settings. These include UMCs, and distributed healthcare settings such as district (teaching) hospitals, ambulatory care settings, and long-term care facilities. The duration of these clerkships varies from a few weeks to several months. UMCs play a coordinating role in medical education in their own region, and are typically responsible for curriculum development and evaluation.

We undertook an exploratory qualitative study, underpinned by a constructivist paradigm. We used individual interviews to understand professionals’ experiences concerning workplace-based learning in distributed healthcare settings.

### Participants

We aimed to include participants involved in the clinical phase of undergraduate medical education from a range of different healthcare settings (maximum variation sampling) [22]. As data collection and analysis proceeded, we purposively selected additional participants (theoretical sampling) [23]. Participants received a letter explaining the purpose of the interview, as well as a brief topic guide to help them prepare. Written informed consent was obtained from all participants.

### Data collection

Semi-structured interviews were conducted by the principal investigator, MV, and were audio-recorded. Interviews took place between August 2020 and September 2021, and averaged 60 min. Most interviews were conducted online due to Covid-related restrictions on face-to-face meetings. We composed an interview guide (Additional file 1) based on a literature review and an expert brainstorming session. We conducted a test interview with a purposively sampled participant to gather feedback on the interview guide. Because of the information value of the data, we decided to include this interview in our sample for analysis. The data collection ended when saturation of themes was reached [24].

### Data analysis

Data collection and analysis took place simultaneously to inform sampling of participants and to enhance the interview guide. Interviews were transcribed verbatim, and anonymized. We used Qualitative Analysis Software Atlas.ti (ATLAS.ti Scientific Software Development GmbH, version 9.1.1) to organize and code the data. We analyzed the data using inductive thematic analysis [25, 26]. Two researchers (MV and RE) independently coded all transcripts. We first extracted and developed concepts from raw data. Next, we identified relationships between the open codes (code list in Additional file 2). After ten interviews, MV and RE constructed a preliminary diagram of code categories to facilitate group discussions with the research team (MV, RE, JK, AL, RL). In group discussions, we identified eight core concepts in the data. We conducted three additional interviews to deepen the our understanding of the identified concepts and the relationships between them. This led to the construction of five themes, which are presented in the Results section. While collecting and analyzing data, MV also wrote memos to help to recognize and expand processes and patterns within the codes [27]. Additional file 3 depicts an approximate overview of the process of qualitative data analysis, showing themes, concepts, and codes.

### Ethical approval

The Ethics Review Board of the Dutch Society for Medical Education granted ethical approval (Nederlandse Vereniging voor Medisch Onderwijs [NVMO]; case no 2020.4.3).

### Reflexivity

MV is the primary investigator; she is a PhD candidate and a medical doctor. AL is a medical doctor, dean of a district teaching hospital, and associate professor of medical education in healthcare networks. RE is an educationalist and a senior researcher. JK is a philosopher and an associate professor of transformative learning. RL is a medical doctor, a professor of health professions education, and director of the educational institute of a university medical center. The authors bring a diverse range of perspectives to the study. MV graduated recently from a medical education program, AL provides insights from the perspective of a practitioner in a distributed setting, RE offers an educational researcher perspective, JK brings a philosophical lens to learning and development, and RL has extensive experience in development and evaluation of medical education and educational policy.

## Results

**Fig. 1:**
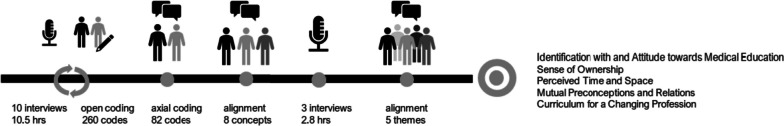
Overview of data collection and analysis

Our research question was: *What are the beliefs, attitudes, and behaviors of professionals concerning workplace-based learning in distributed healthcare settings?* We interviewed 11 physicians (2 internists, 2 pediatricians, 3 general practitioners, 1 public health physician, 1 rehabilitation physician, 1 elderly care specialist, 1 occupational health physician), 1 educationalist, and 1 psychologist/educator. 9 participants were female, 4 were male. The participants were employed in primary care facilities/departments (*n* = 4), district teaching hospitals (*n* = 3), public health facilities (*n* = 3), a university hospital (*n* = 1), a nursing home (*n* = 1), and a rehabilitation center (*n* = 1). Participants were all involved in the clinical phase of undergraduate medical education, and most had a dedicated educational role such as clerkship coordinator or supervisor. Figure 1 provides an overview of data collection and analysis.

### Overview of results

We defined five themes that reflect professionals’ beliefs, attitudes and behaviors towards workplace-based learning in distributed healthcare settings: Identification with and Attitude towards Medical Education, Sense of Ownership, Mutual Preconceptions and Relations, Curriculum for a Changing Profession, and Time and Space. To start to unravel the complexity of workplace-based learning in distributed medical education, we present these themes as distinct, though they do overlap. As participants underlined the significance of their context, the themes relate not only to an individual level, but also to a team and organizational level. We discuss these five themes below and illustrate them with exemplary quotes.

### Identification with and attitude towards medical education

We found that the extent to which workplace-based learning is fostered amidst the pressures of daily healthcare practice hinges on the attitudes and consecutive behaviors of individuals, teams, and organizations. The following quote shows that individual motivation proved to be decisive in facilitating workplace-based learning in a particular setting:

*“When I took on the job here 13 years ago, I was immediately contacted by someone from the GP institute who was in charge of students’ training programs. My predecessor had never felt like it, but I did, so right from the word go we’ve had students here. Let’s just give it a go and see what happens.”*

Similar on an organizational level: if an organization identifies with medical education as a core mission and facilitates it at all levels, this is beneficial to the process of shaping workplace-based learning. An educationalist at a district teaching hospital observed:

*“Perhaps it’s because our hospital has said: we are part of an alliance of collaborating top-clinical hospitals, and so we do training programs. And of course there’s the Board and the Central Training Committee who dare to give people a share of the responsibility; there’s the Central Training Committee who defend people’s interests; and there’s an educationalist to help with the quality care instruments. So there’s a whole institute, education department and all, that enables you to do it. It’s being facilitated.”*

Beliefs about workplace-based learning in practice, and about what being an educator entails, appeared to influence involvement in workplace-based learning. The following quote is from a general practitioner who, despite her extensive educator experience, doubted the added value of her participation in our research:

*“What I told you in my email: when I saw those questions, I was thinking gee, I can’t do any of that. We always just muddle through in this place. That’s not true, of course, but then again it is, to some extent.”*

### Sense of ownership

There were varying degrees to which participants felt ownership over medical education and the curriculum in the different healthcare settings outside the UMC. Participants felt there was some distance between themselves and the curriculum, which involves the risk of eliciting a distant attitude towards workplace-based learning. As an educationalist outside the UMC aptly said:

*“Obviously, I’m not the one deciding what can or cannot be on offer; those decisions are taken by the UMC. We’re actually their subcontractors.”*

Within their own team or organizational contexts, individuals were sometimes up against an environment not focused on workplace-based learning. The following quote shows that despite encountering disinterest from others around her, a physician in a district teaching hospital demonstrates a sense of ownership over workplace-based learning:

*“I remember we were pitching an educational innovation that would take place in our practice [setting] to our medical staff. Half of the people in the room just got up and left. They were just not interested, it costs time. You really have to go the extra mile to snag them. So, one of our medical specialists took it upon herself to personally visit all departments in order to highlight the innovations’ purpose and importance.”*

Priorities laid out by their organization influenced what opportunities participants perceived to be involved in workplace-based learning. In this light, we found that the presence of a dedicated contact, or portfolio holder, for educational affairs at a workplace influenced the sense of ownership of education within that particular context. A physician working in a district teaching hospital voiced her concerns as follows:

*“[sighs] We used to have a director who was very education-minded, but at present we have no education portfolio holder. So, that’s interesting. So [smiles], let’s wait and see, for education is very much the Cinderella of the family, always the very last to be attended to because all the others come first.”*

### Perceived time and space

Participants felt that workplace-based learning was often hindered by the absence of time and space for workplace-based learning. A public health physician described:

*“Partly, this increases the pressure of work in your work environment. We’re not allowed to schedule fewer children when there’s a student involved, but students who see children themselves do need more time, of course. Nor do we have a separate room where they can see a child while I’m seeing another child somewhere else. So this really involves direct supervision, which does take time.”*

Other aspects that appeared to be conditional for workplace-based learning included resources for learners, such as computers and cell phones, and support with dealing with administrative demands that come with being formally recognized as an accredited educational practice by the medical school. A physician working in a specialized primary care facility observed:

*“They also need their own workspaces, you know. You see, we’re not a hospital with plenty of rooms everywhere. When I’m expecting a student, I need to know when they’re coming, I need to arrange a workspace for them and a computer, all that sort of things. (…) Sure, it’s fun, but arranging everything takes tons of time. There’s plenty of thick volumes to plough through, with all sorts of criteria and registrations that must be applied for and I’m thinking: what am I doing it for?”*

### Mutual preconceptions and relations

Mutual perceptions and the quality of relations between individual healthcare professionals, but also between organizations, appeared to influence professionals’ attitudes and behaviors towards workplace-based learning. The following quote outlines how a perceived hierarchical relationship discouraged a general practitioner from pursuing involvement in the medical curriculum:

*“No, I don’t need to know now what the Bachelor’s or whatever looks like. Let’s just leave that in the capable hands of those at the top [of the UMC], shall we?”*

Communication about mutual preconceptions can shift dynamics between different institutions. Talking positively about other professionals or disciplines involved in education, for instance, and acknowledging their legitimate participation can boost the role of educators in workplace-based learning. A general practitioner (GP) mentioned this positive example:

*“How you talk about each other. Before you know it, you’ve said something or shown something that students store in memory, as do patients, by the way. It really cheers me up when a specialist says something like ‘oh, the GP can do this just as well.’ (…) Mutual respect, you know, and communicating it, that’s very important.”*

There also appeared to be negative preconceptions about the quality of workplace-based learning in other institutions. Such perceptions did not appear to help to take optimal DME design as a joint responsibility. A physician working in a UMC remarked:

*“The problem we’ve often had with these clerkships in other places [than the hospital] is that students end up doing nothing at all; public health medicine being a case in point. We’ve been offering it for many years, but in the end students just complain about it being a lost clerkship, where they can do nothing and are not allowed to do anything. (…) I actually feel that you should only do this [training in other places] if there’s actually something for students to learn there.”*

### Curriculum for a changing profession

Participants highlighted the need to align the curriculum with distributed healthcare practice to facilitate workplace-based learning in their setting, to ultimately reach learning outcomes that fit with the changing healthcare profession. They recognize problems herein, for instance with regard to planning and coordination. This educator at a UMC contrasted the benefits of a more centrally implemented curriculum with training in a distributed training landscape:

*“Well, there’s the practical side of things, of course: the good thing about a hospital is that it can process a whole load of students in a fairly structured manner. So from a logistics point of view, this is the easiest way to go.”*

Various interests come into play as professions and professionals aim to have a place in the curriculum. Viewing the contents of a curriculum as an exhaustive list that must cover all topics does not yet demonstrate a changing vision of learning in practice. An occupational physician expressed disappointment about his subject being underappreciated in the curriculum.

*“I lectured for many years. (…) A few years ago, when the curriculum was redrafted, they took occupational medicine out, which I regret. If you want to improve participation, how can you remove it?! Surely occupational medicine is at the core for people who want to work. It’s not all about curative care.”*

It is challenging to reflect on learning for a changing practice, and to determine where and how one can acquire certain knowledge and skills. An educational coordinator working at the primary care department of a UMC said:

*“It’s one of these dilemmas, isn’t it: will you do intramural or extramural training for students? I became a general practitioner in the end, but where did I actually learn to assess an acute abdomen? During my surgery clerkship, I was in the Emergency Department, where we would have someone with abdominal pain coming in about five to ten times a day, and we had to exclude the possibility of it being an acute abdomen. So that’s where I learned it. But in order to acquire a wider and more generalist view, you need to get out of the hospital.”*

And then, merely deciding on a new curricular or didactic approach does not guarantee that it will be a practical and widely accepted way of teaching and learning in practice. A physician in a district teaching hospital who recently started working with Entrustable Professional Activities (EPAs) said:

*“For a student, it’s quite easy to get your scores for making a diagnostic or therapeutic plan, and ka-ching, you’ve earned another EPA. But you might be thinking to yourself, well, you may have done it, say, four times in an asthma patient, you’ve got your EPA, but what does your score signify and what can you really do? It bugs me. (…) We get a lot of input from the department and the doctors, and this is the most instructive. It also helps if you’ve done it for a number of years because then you’ve simply seen a lot of students.”*

## Discussion

In this research, we scrutinized the beliefs, attitudes, and behaviors of professionals concerning workplace-based learning in distributed healthcare settings. We defined five themes: Identification with and attitude towards medical education, Sense of ownership, Perceived time and space, Mutual preconceptions and relations, and Curriculum for a changing profession. Below, we first discuss how our themes relate to existing knowledge from literature of workplace-based learning. Next, we describe tensions and contradictions between practice and education that we identified in our themes, and elaborate on the consequences thereof. Finally, we offer recommendations for practice, and provide suggestions for further research.

The definitions of the themes *Identification with and attitude towards medical education* and *Sense of ownership* are in line with existing knowledge on the challenging position of education as a core task. We recognized that the position of education might be even more challenging in distributed settings than in UMCs, as multiple interviewees from distributed settings, despite their own involvement in education, repeatedly talked about education as if ownership of it belonged more to UMCs. In addition, participants in our study emphasized the importance of establishing a structure and strategy aimed at prioritizing workplace-based learning at both the team and organizational level. These findings correspond to research describing team and organizational structure, strategy, and culture as core components for supporting educator agency, motivation and identity [28–30].

Next, our finding that educators yearn for more *Time and space* for workplace-based learning is also evident from other literature on workplace-based learning [7, 31]. This perceived lack of time and space is frequently described in the context of a competition between education and patient care for the limited time, space and resources in healthcare practice [28, 32]. The lower availability of staff, time, and physical space in smaller distributed settings might add to the challenge of allocating these resources for education, especially when we know that their funding often mostly depends on patient care [9, 33]. To change this, the challenge lies in framing the allocation of budget to the design and quality of workplace learning as an investment in quality of care rather than as an impediment to production efficiency [16, 34, 35].

Where literature describes negative preconceptions about other professionals to hamper collaboration in patient care, according to our participants, the same holds true for collaboration in education [36, 37]. Our findings on how *Mutual relations and preconceptions*, and consequent communication among professionals, students and patients, have their impact on workplace-based learning, are also reflected in literature [38]. Specifically to the DME context, van Schalkwyk et al. also emphasize investing in relations between individuals, teams, and organizations when it comes to implementation of DME on a more overarching level [10].

The theme *Curriculum for a changing profession* reflects participants’ struggles in conceptualizing a curriculum that prepares students for the changing demands of healthcare practice. It is no wonder that they find this challenging, as they discuss the curriculum as an abstract and distant concept, one that belongs to *“those at the top of the UMC”.* As a result, they appear to perceive a disconnect between what they do in practice and the curriculum. Literature and policy documents offer limited help in this regard, as they mainly provide broad guidelines for the future of health professions education, and struggle with how to develop concrete and feasible ways to work with these in practice [7, 39–41].

Zooming out, looking across the themes described from our data, we recognize two almost archetypical and different ways of perceiving the workplace and workplace-based learning in our participants’ responses. As outlined in existing research on workplace-based learning, two distinct frames exist that can collide in practice: those of practice and education [16, 42]. We recognize this clash in our study, for example in the way time and space constraints are talked about. It seems our respondents perceive medical education as detracting from the time and space available for real work. Another example from our study where teaching and work are described as two incompatible entities at the organizational level lies in how respondents clearly described the UMC as the institution for education and learning, and distributed settings as settings for patient care, for work.

When describing the workplace-learning environment as a place where the frames and worlds of learning and working converge, participants in our study operate at the intersection of education and practice. Their position in medical education is often defined in a formal, dedicated educational role in the context of their work environment. This type of role on the border of two domains is also described in the literature as a ‘broker-role’. Brokers are described as individuals who connect multiple worlds through their work across boundaries [43]. In the context of workplace-based learning, we interpret the presence of these formalized roles as a strong sign of the importance placed on education in those settings, and a recognition of the importance of fostering, organizing, and managing that education within the context of clinical practice.

There are, however, two difficulties with the broker role known from the literature. First, broker-roles are often ill-defined. Second, brokers may struggle with their position in-between domains, and the resulting limited visibility, limited recognition, and limited support for the work they do [43, 44]. Our interviewees similarly described challenges in working within teams and contexts not geared towards learning. A telling example of this (described under Sense of Ownership) is the medical specialist who, as a broker, personally visited her colleagues in other departments to reiterate the importance of an educational innovation after they had walked out of the room during a plenary presentation on the subject. We contend that those who play a broker role between the worlds of practice and education in distributed learning workplaces can benefit from gaining recognition among themselves and others of this challenging position they occupy. In addition, we support the direction provided in the literature to further strengthen these important positions by supporting brokers in making their broker activities explicit, and in opening up the conversation about the requirements necessary to undertake their broker role in practice [44–46].

Recognizing, retaining, and strengthening these individuals is crucial, as we find that they play a pivotal role in workplace-based learning in practice. However, respondents’ statements that express uncertainty about their own position and importance (e.g., *“we always just muddle through in this place”*) indicate that they may not always recognize the value of their own contribution, or even downplay it. We advocate for making their valuable, but often implicit and intuitive, contributions to shaping workplace-based learning more explicit. On an overarching level, we suggest that curricular reform should be rooted in practice, revolving around individuals whose involvement is crucial to the success of workplace-based learning. In our view, promising directions include longitudinal integrated clerkships, and co-creation of learning environments with professionals, students, and patients [47–49]. For future research, we encourage research designs that identify, promote, and build from good-practices in workplace-based learning, and designs that are focused on individuals and helping them make an impact in their teams and organizations.

A strength of our study is the variety of healthcare professionals from different types of healthcare settings we spoke to, whose perspectives and experiences offer valuable insights towards our research question. They were all working within the same Education and Training Region in the Netherlands, therefore our findings reflect some of the specific organizational and cultural aspects of this context. This could be perceived as a limitation, however, it aligns with our goal to provide a rich description of our interviewees’ subjective experiences concerning workplace-based learning in distributed settings, and in doing so, to inform others who are engaged in workplace-based learning development in changing healthcare settings. We recognize that conducting most interviews online, necessitated by Covid-related restrictions, could have influenced the interview dynamics. While the virtual format allowed for flexibility and engagement of professionals during a challenging period, potential drawbacks include challenges in capturing nuanced non-verbal cues and the absence of in-person rapport.

## Conclusions

The five themes we identified, namely Identification with and attitude towards medical education, Sense of ownership, Perceived time and space, Mutual preconceptions and relations, and Curriculum for a changing profession, demonstrate aspects relevant to the development of workplace-based learning in distributed healthcare settings. While we underline that it is important to establish a structure and strategy that prioritizes workplace-based learning at both the team and organizational level, our most important finding is that individual professionals’ involvement appeared to be pivotal in the development of workplace-based learning. We suggest to recognize and support these individuals in the difficult position they may occupy as ‘brokers’ on the intersection between education and practice. In addition, we think it is crucial to make their valuable motivation and efforts towards shaping workplace-based learning more explicit. For curriculum development and future research, we recommend prioritizing initiatives that build from what is already proving it’s worth in practice: to start from good-practices in workplace-based learning, and to engage and involve dedicated individuals in distributed healthcare settings.

### Supplementary Information

**Additional file 1.** Interview guide.

**Additional file 2.** List of codes after axial coding.

**Additional file 3.** Tables depicting an approximate overview of the process of qualitative data analysis, showing themes, concepts, and codes.

## Data Availability

The data (anonymized interview transcripts) analyzed during the current study are available from the corresponding author on reasonable request.

## Abbreviations

DME: Distributed Medical Education
EPA: Entrustable Professional Activity
GP: General Practitioner
UMC: University Medical Center
