# Supplementary material for: Exploring workplace-based learning in distributed healthcare settings: a qualitative study
Source: BMC Med Educ. 2024 Jan 22;24:78. doi: 10.1186/s12909-024-05053-6 (PMC10804752; doi:10.1186/s12909-024-05053-6)
Supplement: Supplementary file 1 — Additional file 1. Interview guide. [file 12909_2024_5053_MOESM1_ESM.pdf]

# Additional file 1: Interview guide / topic list

What comes to mind when I ask you about workplace-based learning at your workplace?

**General topic list:**

- Organization / Leadership:
  - Relation/communication with the UMC and other institutions involved in medical education
  - Educational vision, strategy, structure, culture
- Curriculum
  - Implementation of the curriculum at your workplace
  - Learning outcomes
  - Duration of clerkships at your workplace
- Community
  - Patient involvement in learning
  - Community healthcare needs reflected in learning
- Facilities
  - Allocation of resources
  - Preconditions for workplace-based learning
  - Support, time, space for learning
- Staff
  - Recognition, support, opportunities for educator development
  - Dedicated educational roles
- Students
  - Selection of students
  - Students’ expectations of clerkships
