# Supplementary material for: Exploring workplace-based learning in distributed healthcare settings: a qualitative study
Source: BMC Med Educ. 2024 Jan 22;24:78. doi: 10.1186/s12909-024-05053-6 (PMC10804752; doi:10.1186/s12909-024-05053-6)
Supplement: Supplementary file 2 — Additional file 2. List of codes after axial coding. [file 12909_2024_5053_MOESM2_ESM.pdf]

Additional file 2: List of codes after axial coding

Codes are listed in alphabetical order, numbers are referred to in Appendix C.

1. Administrative) support / staff needed
2. Advertising your own profession
3. Aligining education and healthcare
4. Aligning curriculum and practice
5. Aligning education and healthcare demands
6. Assessment
7. Being invested in learning/education
8. Capacity
9. Clerkships' image to students
10. Collaboration
11. Community based education
12. Community of educators
13. Continuity in supervision
14. Coordination is needed
15. Cross-pollination
16. Curriculum is difficult to change
17. Dedicated clerkship coordinator/educator
18. Dedicated individuals vs. teamwork
19. Dedicated residency coordinator/educator
20. Departmental culture
21. Departmental strategy
22. Departmental structure
23. District teaching hospitals' role in education development
24. Diverse role models
25. Duration of clerkships
26. Edcuator motivation
27. Education of residents vs. of medical students
28. Educator quality
29. Exposure
30. Exposure to students
31. Facilities
32. Faculty development

33. Flexibility
34. Flexible/personalized learning goals for students
35. Formal / informal learning
36. Fragmentation in healthcare
37. Funding structures
38. Increasing the number of learning environments offers more opportunities for learning
39. Involving new/different professionals in education is an opportunity for change
40. Knowledge about the curriculum / learning goals
41. Learnability of students
42. Learning climate
43. Learning goals / didactics
44. Logistical challenges
45. Longitudinal workplace-based learning
46. More/Broader opportunities for student development
47. New types of workplace-based learning
48. Opportunities for a culture change
49. Opportunities for new or different types of healthcare
50. Opportunities for practice based teaching/education
51. Opportunities of rich, authentic learning environments
52. Opportunities of smaller heatlchare teams
53. Organizational aspects of the curriculum
54. Organizational culture
55. Organizational strategy
56. Organizational structure
57. Patient involvement in learning
58. Patients’ role in education
59. Production / Efficiency
60. Professionalizing healthcare sectors/domains through educational involvement
61. Quality assurance (curriculum)
62. Recognition for educators

63. Relations UMC/distributed practices
64. Requirements for graduate physicians
65. Residents’ role in medical students’ education
66. Role description needed
67. Role integration
68. Role model / Example
69. Role of professionals associations
70. Sharing educational responsibilities/tasks within teams
71. Short lines of communication between students and educators
72. Short lines of communication within organizations/practices
73. Steering curriculum
74. Student selection
75. Student wellbeing
76. Students’ experience in vs exposure to a certain topic
77. Timing within learning continuum
78. UMC / distributed practices
79. Views / perspective (on healthcare / education)
80. What do/should students learn where?
81. Who can be an educator/assessor?
82. Workplace-based learning
