# Supplementary material for: Exploring workplace-based learning in distributed healthcare settings: a qualitative study
Source: BMC Med Educ. 2024 Jan 22;24:78. doi: 10.1186/s12909-024-05053-6 (PMC10804752; doi:10.1186/s12909-024-05053-6)
Supplement: Supplementary file 3 — Additional file 3. Tables depicting an approximate overview of the process of qualitative data analysis, showing themes, concepts, and codes. [file 12909_2024_5053_MOESM3_ESM.pdf]

Additional file 3: Tables depicting an approximate overview of the process of qualitative data analysis, showing themes, concepts, and codes.

Tables A and B are meant to provide an approximate overview of the process of our qualitative data analysis. It offers a simplification of how our understanding of the data evolved throughout the iterative process, going from codes to concepts (Table A), and subsequently from concepts to themes (Table B) that help to tell the story of our results.

Table A: Table A shows the clustering of qualitative data into 8 core concepts, with a description of the concept, and an overview of the codes included in the concept. Numbers in 'Codes' column correspond to code numbers in Additional file 2.

Concepts	Description	Codes
Responsibility	Feelings of responsibility towards educating future physicians in general, and more specifcally for workplace-based learning in one's own healthcare practice.	2,7,13,14,17,18,19,22,23,28,32,37,40,41,43,60,61,66, 69,70,78,79,80,81
Relations	Relations and collaborations between individuals, teams, and organizations involved in education.	10,11,12,14,15,18,23,33,36,38,42,48,52,54,57,58,62, 63,65,70,71,72,78,81
Agency	Individuals', teams' or organization's decision and consecutive actions to be involved in workplace-based learning.	1,2,3,8,21,22,31,32,35,37,40,41,47,48,49,51,52,53,55, 59,60,67,69
Insecurity	Dealing with insecurity about one's educator role, and about changes in medical education and practice.	4,5,24,28,34,35,43,50,53,64,66,75,76,77,81
Trust / Entrustment	Dealing with entrustment / trust in assessment of students.	6,14,34,42,57,58,64,74,75,76,77,80,81,82
Openness	Openness towards workplace-based learning, and towards transforming healthcare practice for learning.	14,16,23,24,33,39,42,46,47,49,54,62,68,70,73,75,82
Frames / Beliefs	Prevailing frames and beliefs about (workplace-based) learning and education.	2,3,5,6,8,9,14,15,16,18,20,21,22,25,26,27,28,29,30,34, 36,37,42,43,44,45,47,48,49,50,51,54,56,59,62,67,68, 73,74,77,79,80,81,82
Prerequisites	Logistics that are needed to support workplace-based learning.	1,8,14,21,22,25,31,33,37,44,55,56,59

Table B: Table B shows the 5 themes that were constructed out of qualitative data, and provides an overview of the concepts included in the themes

**Themes**	**Concepts**
Identification With and Attitude towards Medical Education	Responsibility, Agency, Insecurity, Frames / Beliefs
Sense of Ownership	Responsibility, Agency, Frames / Beliefs
Perceived Time and Space	Prerequisites
Mutual Preconceptions and Relations	Relations, Openness, Trust / Entrustment
Curriculum for a Changing Profession	Trust / Entrustment, Openness, Frames / Beliefs
